# Intolerance of Uncertainty, Job Satisfaction and Work Performance in Turkish Healthcare Professionals: Mediating Role of Psychological Capital

**DOI:** 10.3389/ijph.2024.1607127

**Published:** 2024-06-24

**Authors:** Murat Yıldırım, Zafer Güney Çağış, Juan Gómez-Salgado

**Affiliations:** ^1^ Department of Psychology, Faculty of Arts and Sciences, Ağrı İbrahim Çeçen University, Ağrı, Türkiye; ^2^ Department of Social and Educational Sciences, Lebanese American University, Beirut, Lebanon; ^3^ Department of Sociology, Social Work and Public Health, Faculty of Labour Sciences, University of Huelva, Huelva, Spain; ^4^ Safety and Health Postgraduate Programme, Universidad Espíritu Santo, Guayaquil, Ecuador

**Keywords:** psychological capital, intolerance of uncertainty, job satisfaction, work performance, healthcare professionals

## Abstract

**Objective:** Psychological capital refers to internal resources including self-efficacy, hope, optimism and resilience to overcome adverse life events. The current study sought to examine the mediating role of psychological capital in the relationship between intolerance of uncertainty and job satisfaction and work performance in healthcare professionals.

**Methods:** Participants were 302 healthcare professionals [48% females; *M(SD)*_age_ = 34.0 (7.5)] and completed measures of intolerance of uncertainty, psychological capital, work performance, and job satisfaction.

**Results:** The findings indicated that intolerance of uncertainty was negatively correlated with psychological capital, work performance, and job satisfaction, whereas psychological capital was positively correlated with job satisfaction and work performance. More importantly, the findings revealed that these relationships were mediated by psychological capital.

**Conclusion:** The results provide several contributions that help to understand the role of psychological capital in the relationship between intolerance to uncertainty and job satisfaction and work performance.

## Introduction

The precautions taken during COVID-19 and the uncertainty about when and how the virus will end, being infected and the loss of a close person caused psychological problems including loneliness, fear, stress, depression, and anxiety [1–5]. These difficulties have been widely experienced by individuals across different populations [6–9]. However, healthcare workers, in particular, have been disproportionately affected by the negative psychological consequences of the pandemic [10]. Compared to the general population, healthcare workers have been found to experience higher levels of anxiety, somatization, insomnia, and depressive symptoms [11]. Similarly, previous research has revealed that healthcare workers experience high levels of emotional burnout and depersonalisation [12], depression, anxiety and sleep problems [13, 14], and decreased job satisfaction [15]. In addition, during this process, they have faced situations that would affect their performance such as excessive attention, concentration, responsibility, workload and long or irregular working hours [16].

Intolerance of uncertainty (IU) is a dysfunctional fear that underlies emotional difficulties and causes anxiety in situations when a lack of knowledge is perceived [17]. IU disrupts functional emotional and cognitive processes [18]. Therefore, IU causes various negative consequences including anxiety [19], depression, and post-traumatic stress disorders [20]. Individuals with high IU levels are at higher risk of developing psychological problems such as stress when they are exposed to various problems in daily life [21]. IU is correlated with concern about COVID-19 [22], fear of COVID-19 [23], depression [24], sleep problems [25], and predicted mental wellbeing [26] and mediated the relationship between social isolation and psychological distress [27] during the pandemic. In addition, a previous study has reported that there is an inverse link between IU and job satisfaction, although not during the pandemic [28].

Job satisfaction is a critical factor that enables healthcare professionals to provide efficient healthcare services [29]. Job satisfaction is a positive emotional response that reflects the degree to which people find it satisfying [30]. Job satisfaction can protect employees from stress factors [31]. Job satisfaction is positively associated with high self-confidence and productivity [32], and low anxiety and stress [33] in employees. More importantly, high job satisfaction creates a positive environment in the workplace, increases productivity and reduces job stress [30, 34]. In addition, it is negatively correlated with turnover intention [35, 36] and positively related to work performance [37].

### Psychological Capital as a Mediator

Psychological capital (PsyCap) refers to internal resources to overcome adverse life events. In other words, psychological capital is a psychological construct that consists of individuals’ levels of self-efficacy, hope, optimism, and resilience, and that protects individuals against various psychological problems [38]. PsyCap is positively associated with wellbeing [39] and reduces the negative impact of stressful life events [40]. It significantly affects the work-related behaviours of employees [41]. Earlier studies revealed that PsyCap is positively correlated with job satisfaction, and work performance [42–44], and it is an important source of psychological support at work in difficult life events [45]. Indeed, PsyCap is related to mental health in healthcare workers [46] and can alleviate the negative psychological effects of COVID-19 [47, 48]. More importantly, Liu et al. [49] have revealed the mediating role of PsyCap in the relationship between occupational stress and depressive symptoms among Chinese physicians. Similarly, Tian et al. [50] have also found that PsyCap has a mediating role in the relationship between occupational stress and fatigue in healthcare professionals. More recently, Mubarak et al. [48] have revealed the mediating role of PsyCap in the relationship between public health education and fear of COVID-19 in nurses, while Yıldırım et al. [51] have demonstrated that PsyCap acts as a mediator in the relationship between fear of COVID-19 and intolerance of uncertainty and positive future expectations in healthcare professionals during the COVID-19 pandemic.

### Present Study

**FIGURE 1:**
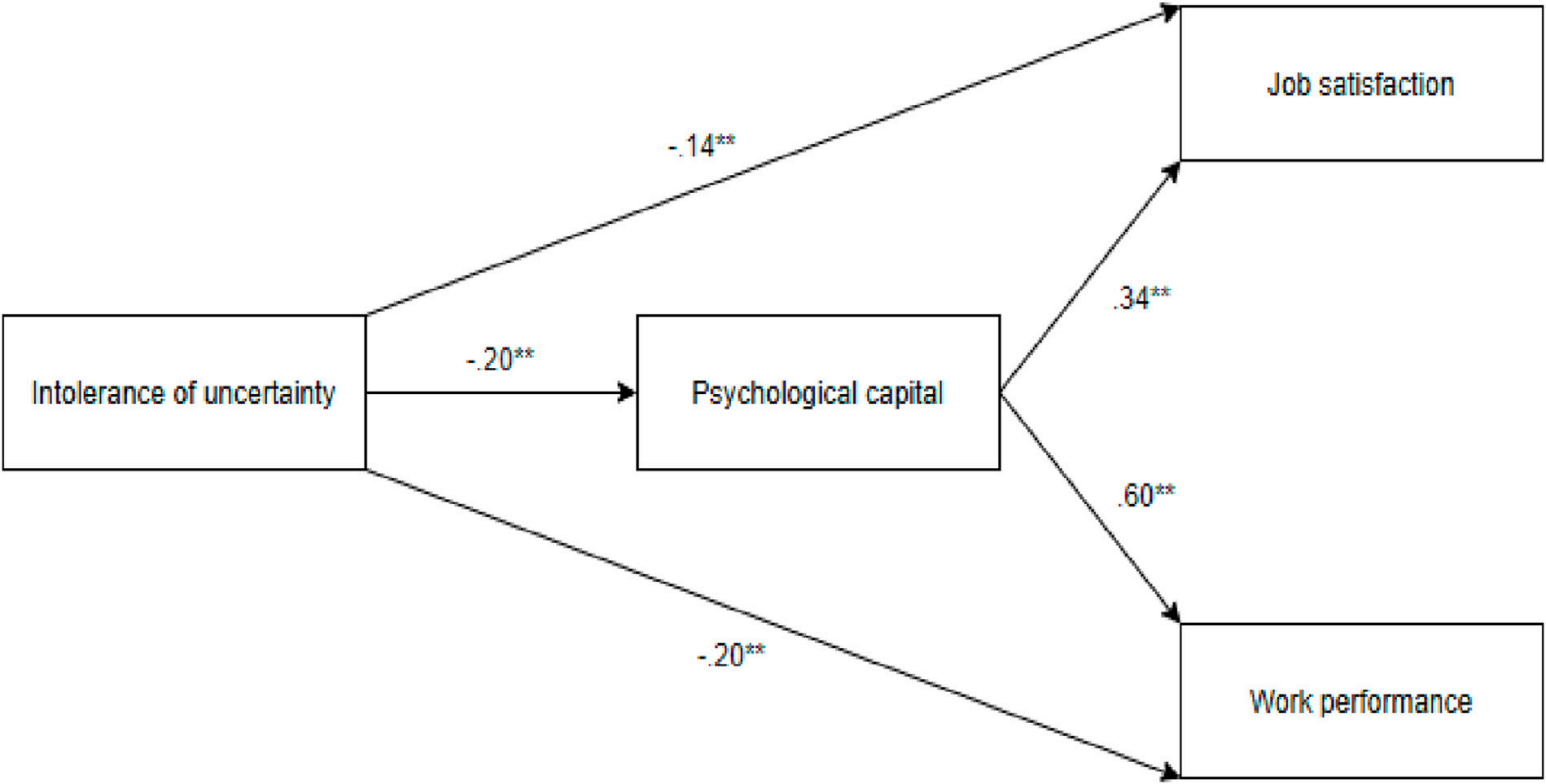
The proposed hypothesised model depicting the mediating impact of psychological capital in the association between intolerance of uncertainty and work performance and job satisfaction (Türkiye, 2024).

Job satisfaction and work performance among healthcare professionals are vital for maintaining uninterrupted and high-quality health services during COVID-19. While numerous studies have explored the psychological effects of COVID-19 on healthcare workers [47, 51–53], to the best of our knowledge, no research has specifically examined the relationship between IU, work performance, and job satisfaction among Turkish healthcare professionals during the pandemic. The main purpose of this study is to reveal the mediating role of PsyCap in the relationship between IU, job satisfaction and work performance in a sample of healthcare professionals in Turkey. To end that, the following hypotheses were generated: 1) IU is negatively correlated with PsyCap, job satisfaction, and work performance; 2) PsyCap is positively correlated with job satisfaction and work performance; 3) PsyCap mediates the association between IU and job satisfaction; 4) PsyCap mediates the association between IU and work performance (see Figure 1).

## Methods

### Procedure

The snowball sampling method was used to recruit participants through secure software. Healthcare professionals working at various hospitals in Turkey were invited to the study through applications such as SMS, WhatsApp and e-mail. Healthcare professionals willing to participate in the study were then asked to invite their colleagues to participate in the study. Afterwards, a link containing detailed study information, an informed consent form and scales was sent to the participants. Before completing the questionnaires, participants were required to provide their informed consent. The participants were assured that their responses would be treated with strict confidentiality and anonymity. The study was conducted after obtaining ethical approval from (Blinded for review) University Research Ethics Board.

### Participants

351 healthcare professionals were approached and 302 of them were willing to participate in the study. Thus, the study sample consisted of 302 healthcare workers, with 145 (48%) identified as females and 157 (52%) as males. The mean age of participants was 34.0 years (*SD* = 7.5, range = 20–61). The majority of the participants, accounting for 92.7% of the sample, had completed a university degree, while the remaining 7.3% of the participants had completed a high school diploma.

### Measures

#### Uncertainty Intolerance Scale

The Uncertainty Intolerance Scale (IUS-12) is a 5-point Likert-type (1 = *strongly disagree* to 5 = *strongly agree*) scale consisting of 12 items (e.g., “When it’s time to act, uncertainty paralyses me”) [54]. The scale is used to evaluate the anxiety levels of the participants in uncertain situations. High scores indicate high anxiety. Sarıçam et al. [55] conducted an assessment of the psychometric properties of the Turkish version of the IUS-12. In this study, Cronbach’s alpha was 0.87.

#### Psychological Capital Questionnaire

Psychological Capital Questionnaire (PCQ-12) consists of 4 sub-dimensions: optimism, hope, resilience and self-efficacy [56]. The scale consists of 12 items (e.g., “I can think of many ways to reach my current goals”). Each item is rated on a 5-point scale, varying from 1 (*strongly disagree*) to 5 (*strongly agree*). The PCQ-12 was validated in Turkish by Çağış and Yıldırım [47]. In this study, Cronbach’s alpha internal reliability of PCQ-12 was 0.85.

#### Job Satisfaction Scale

To assess the job satisfaction of the participants, we used the Job Satisfaction Scale developed by Brayfield and Rothe [57] and a short version prepared by Judge et al. [58]. The scale consists of 5 items (e.g., “Each day of work seems like it will never end”) with scoring based on a 5-point Likert-type scale, ranging between 1 (*strongly disagree*) and 5 (*strongly agree*). A total score was created after computing reverse-coded items with higher scores indicating a high level of job satisfaction. The scale was adapted into the Turkish language by Çöl [59]. In this study, Cronbach’s alpha for the scale was 0.86.

#### Work Performance Scale

To evaluate the performance of the employees, the Performance Scale developed by Karakum [60] was used. The scale was developed using the contextual performance scale developed by Borman and Motowidlo [61] and the task performance scales developed by Befort and Hattrup [62]. The scale consists of 11 statements (e.g., “I produce high-quality work”) and each item is answered based on a 5-point Likert-type scale (1 = *strongly disagree* to 5 = *strongly agree*). Work performance is evaluated by the average of 11 statements. A high score indicates high performance. In this study, Cronbach’s alpha internal reliability of the scale was 0.90.

### Data Analysis

Descriptive statistics such as mean and standard deviation were reported for each variable of this study. Skewness and kurtosis statistics alongside their cut-off points were utilised to investigate the normality assumption. Pearson correlation coefficients were computed to investigate the relationships between the analysed variables. Employing the PROCESS macro for SPSS version 3.4, we carried out the mediation analysis with Model 4. The findings from the mediation model were presented using the unstandardised path estimate (Coeff), standardized path estimate (β), and squared-multiple correlations (*R* ^2^). In addition, a bootstrapping procedure with 10,000 resamples was run to calculate 95% confidence intervals (CI) for indirect effect [63, 64]. The analysis was performed utilising SPSS version 26.

## Results

As seen in Table 1, the preliminary results showed that all study variables had normal distribution based on the criterion ≤ |1| (skewness range = −0.59 to −0.21 and kurtosis range = −0.24–0.70) and strong internal consistency reliability with this sample (α range = 0.85–0.90). Pearson correlation analysis indicated a small to a large correlation between the variables of this study. IU was negatively related to job satisfaction and PsyCap. Work performance was positively related to job satisfaction and PsyCap. Furthermore, job satisfaction was positively related to PsyCap (see Table 1).

**TABLE 1:** The results of descriptive statistics, and correlation analysis (Türkiye, 2024).

Variable	Descriptive statistics				Correlations			
	α	Mean (SD)	Skewness	Kurtosis	1	2	3	4
1. Intolerance of uncertainty	0.87	38.06 (8.82)	−0.39	0.05	—	−0.08	−0.21**	−0.20**
2. Work performance	0.9	40.75 (7.46)	−0.5	0.7		—	0.39**	0.56**
3. Job satisfaction	0.86	15.89 (4.55)	−0.21	−0.24			—	0.37**
4. Psychological capital	0.85	41.2 (6.91)	−0.59	0.59				—

** *p* < 0.01.

Most importantly, we investigated whether PsyCap mediated the effect of IU on job satisfaction and work performance. A summary of the results concerning this analysis is presented in Table 2 and Figure 1. The findings demonstrated that IU significantly predicted PsyCap (β = −0.20, *p* < 0.001), accounting for 4% of the total variance in PsyCap. IU (β = −0.14, *p* < 0.001) and PsyCap (β = 0.34, *p* < 0.001) significantly predicted job satisfaction by explaining 16% of the total variance in job satisfaction. In addition, IU (β = −0.20, *p* < 0.001) and PsyCap (β = 0.60, *p* < 0.001) significantly predicted work performance by accounting for 35% of the total variance in work performance. Moreover, the bootstrapped confidence interval analysis for the indirect effect of IU on job satisfaction (effect = −0.04, 95% CI [−0.06, −0.01]) and work performance (effect = −0.10, 95% CI [−0.17, −0.04]) via PsyCap was significant as reported in Table 3. These results suggest that PsyCap partially mediated the impact of IU on job satisfaction and work performance. The great part of the job satisfaction and the job performance variance was explained by the model.

**TABLE 2:** The unstandardised coefficients for the mediation model (Türkiye, 2024).

	Consequent			
	*M* (psychological capital)			
Antecedent	Coeff.	*SE*	*t*	*p*
*X* (Intolerance of uncertainty)	−0.16	0.04	−3.55	<0.001
Constant	47.12	1.71	27.55	<0.001
	*R* ^2^ = 0.04 *F* = 12.62; *p* < 0.001			

	*Y*_*1*_ (Job satisfaction)			
Antecedent	Coeff.	*SE*	*t*	*p*
*X* (Intolerance of uncertainty)	−0.07	0.03	−2.62	<0.001
*M* (Psychological capital)	0.23	0.04	6.38	<0.001
Constant	9.39	1.67	4.77	<0.001
	*R* ^2^ = 0.16 *F* = 28.20; *p* < 0.001			

	*Y*_*2*_ (Work performance)			
Antecedent	Coeff.	*SE*	*t*	*p*
*X* (Intolerance of uncertainty)	−0.17	0.04	−4.26	<0.001
*M* (Psychological capital)	0.65	0.05	12.84	<0.001
Constant	7.60	2.82	2.69	<0.001
	*R* ^2^ = 0.35 *F* = 83.96; *p* < 0.001			

Note. Number of bootstrap samples = 10,000; *SE*, standard error; Coeff, unstandardised coefficients; *X*, independent variable; *M*, mediator variable; *Y*, outcome variable.

**TABLE 3:** Standardized indirect effects (Türkiye, 2024).

Paths	Effect	*SE*	BootLLCI	BootULCI
Intolerance of uncertainty–>Psychological capital–>Job satisfaction	−0.04	0.01	−0.06	−0.01
Intolerance of uncertainty–>Psychological capital–> work performance	−0.10	0.03	−0.17	−0.04

## Discussion

In the current study, we sought to reveal the predictors of job satisfaction and work performance of healthcare workers during COVID-19. For this purpose, we investigated the mediating role of PsyCap in the relationship between IU, and job satisfaction and work performance of healthcare workers. As far as we know, there is no study examining these relationships among healthcare workers during the pandemic. For this reason, the current research provided important information about the factors affecting the job satisfaction, and work performance of healthcare workers.

The results of the analysis confirmed the hypotheses of the research. First of all, correlation analyses showed that IU was negatively correlated with job satisfaction. These results support previous study results [28] indicating that IU is negatively correlated with job satisfaction, although not conducted in the context of the pandemic and healthcare professionals. In addition, previous researchers have found that IU is associated with increased turnover intention and burnout [65]. In this respect, the current research is consistent with antecedent studies indicating the negative impact of IU in the organizational field. This result reveals that healthcare workers with high IU are likely to have low job satisfaction levels, especially during stressful life events such as pandemics. Similarly, the correlation results indicated that PsyCap was positively associated with job satisfaction, and work performance, which is in line with earlier research findings [42, 44]. This suggests that, unlike IU, healthcare workers with high levels of PsyCap may experience high job satisfaction and work performance.

More importantly, we investigated the mediating role of PsyCap in the relationship between IU, job satisfaction and work performance in healthcare workers during the pandemic. These results elucidate the relationship between IU and work-related outcomes by demonstrating that low levels of IU are associated with higher PsyCap, which in turn positively impacts job satisfaction and work performance. This suggests that the negative effects of high IU on these outcomes may be mediated by a reduction in PsyCap, highlighting the importance of psychological resources in mitigating the adverse effects of IU on job satisfaction and performance. There are almost no studies on the effects of IU in the organizational field. Although IU is associated with job satisfaction outside the sample of healthcare workers [28], it is not clear whether this relationship can be confirmed among healthcare workers and the psychological mechanisms between this relationship. However, consistent with our assumptions, PsyCap significantly mediated the association between IU and job satisfaction and work performance. This indicates that PsyCap may decrease the negative impact of IU on job satisfaction and work performance. Thus, the findings are in line with the findings of antecedent research indicating that PsyCap can have a protective effect against negative experiences of healthcare workers during COVID-19 [47, 48, 51, 52].

### Contributions

Notwithstanding the acknowledged limitations, our study has important value in advancing the existing literature by providing evidence on the potential role of PsyCap as a protective mechanism in mitigating the adverse impact of IU on job satisfaction and work performance among healthcare workers, particularly during periods of heightened demand for healthcare services, such as pandemics. The findings of the present study revealed that the decrease in PsyCap was found to be a mediating mechanism, linking the elevated levels of IU to reduced levels of both job satisfaction and work performance among the participants. These results suggest that higher levels of IU may contribute to a decline in individuals’ PsyCap, subsequently impacting their job-related outcomes, underscoring the importance of understanding the associations between IU, PsyCap, and work-related outcomes in organizational settings. Our findings highlight the significance of PsyCap in alleviating the negative effects of stressful and demanding situations that healthcare professionals encounter, enabling them to maintain their job satisfaction and work efficiency amidst the challenges posed by pandemics. Moreover, these findings offer valuable evidence on the design and implementation of targeted interventions aimed at bolstering the PsyCap and work-related outcomes of healthcare professionals during such critical life events as pandemics. Understanding the potential buffer effects of PsyCap on job satisfaction and work performance can inform the development of strategies and support systems that empower healthcare workers to cope effectively with the unprecedented demands and pressures they face during crises. By promoting psychological resources and bolstering PsyCap, healthcare institutions can optimise the delivery of quality healthcare services, even in the face of challenging and uncertain circumstances.

### Limitations

One limitation of this study is its reliance on a non-representative sample of Turkish healthcare workers, which restricts the generalizability of the findings both within Turkey and to other countries or cultural contexts. Differences in healthcare systems and cultural attitudes may affect the applicability of the results. Future research should include diverse samples from various occupational settings and cultures to enhance the generalizability of the conclusions. Another limitation concerns the use of cross-sectional data to examine the mediating role of PsyCap between healthcare workers’ IU and job satisfaction and work performance. Longitudinal data can be employed in subsequent studies to assess work-related outcomes among healthcare workers over time. Furthermore, the use of self-report questionnaires in this study raises potential concerns regarding the influence of social desirability and common method bias on the results. To mitigate these biases, future research should incorporate multiple data sources (e.g., peer reports and objective measures) and diverse methods (e.g., interviews and observations) for data collection. Moreover, subsequent research should consider including additional personal and contextual factors such as working hours and day-night shifts, when analysing the direct and indirect associations between IU, PsyCap, job satisfaction, and work performance. Finally, the essence of mediational analyses is to provide evidence about the nature of relationships between variables, including their directional influence. Hayes [63] argues that testing mediation models is also feasible in non-longitudinal studies. While our proposed model implies a directionality of causality by placing work performance and job satisfaction at the end of our proposed model, this is purely hypothetical and may not reflect real-life dynamics. To accurately test the directionality of causality between these variables, future research should employ longitudinal or experimental studies.

## Data Availability

The datasets generated and/or analysed in the present study can be obtained from the corresponding author upon reasonable request.

## References

[1] Bhuiyan AI Sakib N Pakpour AH Griffiths MD Mamun MA. COVID-19 Related Suicides in Bangladesh Due to Lockdown and Economic Factors: Case Study Evidence From media Reports. Int J Ment Health Ad (2020) 19(6):2110–5. 10.1007/s11469-020-00307-y PMC7228428 32427168

[2] Garfin DR Silver RC Holman EA. The Novel Coronavirus (COVID-2019) Outbreak: Amplification of Public Health Consequences by Media Exposure. Health Psychol (2020) 39(5):355–7. 10.1037/hea0000875 32202824 PMC7735659

[3] Mukhtar S. Mental Health and Psychosocial Aspects of Coronavirus Outbreak in Pakistan: Psychological Intervention for Public Mental Health Crisis. Asian J Psychiatry (2020) 51:102069. 10.1016/j.ajp.2020.102069 PMC7161472 32344331

[4] Yıldırım M Çiçek İ. Fear of COVID-19 and Smartphone Addiction Among Turkish Adolescents: Mitigating Role of Resilience. Fam J (2022):106648072211395. 10.1177/10664807221139510

[5] Yıldırım M Şanlı ME. Psychometric Properties of the Turkish Version of the COVID-19 Impact Scale in University Students. JOSEP (2023) 3(1):22–33. 10.47602/josep.v3i1.34

[6] Chirico F Afolabi AA Ilesanmi OS Nucera G Ferrari G Szarpak L Workplace Violence Against Healthcare Workers During the COVID-19 Pandemic: A Systematic Review. J Health Soc Sci (2022) 7(1):14–35. 10.19204/2022/WRKP2

[7] Hu J Ye B Yildirim M Yang Q. Perceived Stress and Life Satisfaction During COVID-19 Pandemic: The Mediating Role of Social Adaptation and the Moderating Role of Emotional Resilience. Psychol Health Med (2023) 28(1):124–30. 10.1080/13548506.2022.2038385 35139700

[8] Yıldırım M Arslan G Ahmad Aziz I. Why Do People High in COVID-19 Worry Have More Mental Health Disorders? The Roles of Resilience and Meaning in Life. Psychiatr Danub (2020) 32(3-4):505–12. 10.24869/psyd.2020.505 33370760

[9] Yıldırım M Kaynar Ö Arslan G Chirico F. Fear of COVID-19, Resilience, and Future Anxiety: Psychometric Properties of the Turkish Version of the Dark Future Scale. J Pers Med (2023) 13(4):597. 10.3390/jpm13040597 37108983 PMC10143929

[10] Ekingen E Teleş M Yıldız A Yıldırım M. Mediating Effect of Work Stress in the Relationship Between Fear of COVID-19 and Nurses' Organizational and Professional Turnover Intentions. Arch Psychiatr Nurs (2023) 42:97–105. 10.1016/j.apnu.2022.12.027 36842836 PMC9806922

[11] da Silva Neto RM Benjamim CJR de Medeiros Carvalho PM Neto MLR. Psychological Effects Caused by the COVID-19 Pandemic in Health Professionals: A Systematic Review With Meta-Analysis. Prog Neuropsychopharmacol Biol Psychiatry (2021) 104:110062. 10.1016/j.pnpbp.2020.110062 32771337 PMC7409979

[12] Giusti EM Pedroli E D'Aniello GE Stramba Badiale C Pietrabissa G Manna C The Psychological Impact of the COVID-19 Outbreak on Health Professionals: A Cross-Sectional Study. Front Psychol (2020) 11:1684. 10.3389/fpsyg.2020.01684 32754102 PMC7366071

[13] Badahdah AM Khamis F Al Mahyijari N. Sleep Quality Among Health Care Workers During the COVID-19 Pandemic. J Clin Sleep Med (2020) 16(9):1635. 10.5664/jcsm.8624 32515347 PMC7970600

[14] Lai J Ma S Wang Y Cai Z Hu J Wei N Factors Associated With Mental Health Outcomes Among Health Care Workers Exposed to Coronavirus Disease 2019. JAMA Netw Open (2020) 3(3):e203976. 10.1001/jamanetworkopen.2020.3976 32202646 PMC7090843

[15] Leskovic L Erjavec K Leskovar R Vukovic G. Burnout and Job Satisfaction of Healthcare Workers in Slovenian Nursing Homes in Rural Areas During the COVID-19 Pandemic. Ann Agric Environ Med (2020) 27:664–71. 10.26444/aaem/128236 33356076

[16] Yáñez-Araque B Gómez-Cantarino S Gutiérrez-Broncano S López-Ruiz VR. Examining the Determinants of Healthcare Workers’ Performance: A Configurational Analysis During COVID-19 Times. IJERPH (2021) 18(11):5671. 10.3390/ijerph18115671 34070684 PMC8198787

[17] Fergus TA. A Comparison of Three Self-Report Measures of Intolerance of Uncertainty: An Examination of Structure and Incremental Explanatory Power in a Community Sample. Psychol Assess (2013) 25(4):1322–31. 10.1037/a0034103 23937537

[18] Yook K Kim KH Suh SY Lee KS. Intolerance of Uncertainty, Worry, and Rumination in Major Depressive Disorder and Generalized Anxiety Disorder. J Anxiety Disord (2010) 24(6):623–8. 10.1016/j.janxdis.2010.04.003 20439149

[19] Morriss J Macdonald B Van Reekum CM. What Is Going on Around Here? Intolerance of Uncertainty Predicts Threat Generalization. PLoS One (2016) 11(5):e0154494. 10.1371/journal.pone.0154494 27167217 PMC4864232

[20] Hollingsworth DW Gauthier JM McGuire AP Peck KR Hahn KS Connolly KM. Intolerance of Uncertainty Mediates Symptoms of PTSD and Depression in African American Veterans With Comorbid PTSD and Substance Use Disorders. JBP (2018) 44(7):667–88. 10.1177/0095798418809201

[21] Oglesby ME Boffa JW Short NA Raines AM Schmidt NB. Intolerance of Uncertainty as a Predictor of Post-Traumatic Stress Symptoms Following a Traumatic Event. J Anxiety Disord (2016) 41:82–7. 10.1016/j.janxdis.2016.01.005 26803928

[22] Wheaton MG Messner GR Marks JB. Intolerance of Uncertainty as a Factor Linking Obsessive-Compulsive Symptoms, Health Anxiety and Concerns About the Spread of the Novel Coronavirus (COVID-19) in the United States. JOCRD (2021) 28:100605. 10.1016/j.jocrd.2020.100605 33251098 PMC7681070

[23] Deniz ME. Self-Compassion, Intolerance of Uncertainty, Fear of COVID-19, and Well-Being: A Serial Mediation Investigation. Pers Individ Differ (2021) 177(4):110824. 10.1016/j.paid.2021.110824 PMC7945866 33723469

[24] Seco Ferreira DC Oliveira WL Costa Delabrida ZN Faro A Cerqueira-Santos E. Intolerance of Uncertainty and Mental Health in Brazil During the COVID-19 Pandemic. Suma Psicol (2020) 27(1):62–9. 10.14349/sumapsi.2020.v27.n1.8

[25] Voitsidis P Gliatas I Bairachtari V Papadopoulou K Papageorgiou G Parlapani E Insomnia During the COVID-19 Pandemic in a Greek Population. Psychiatry Res (2020) 289:113076. 10.1016/j.psychres.2020.113076 32434093 PMC7217074

[26] Satici B Saricali M Satici SA Griffiths MD. Intolerance of Uncertainty and Mental Wellbeing: Serial Mediation by Rumination and Fear of COVID-19. Int J Ment Health Ad (2020) 20:2731–42. 10.1007/s11469-020-00305-0 PMC7228430 32427165

[27] Smith BM Twohy AJ Smith GS. Psychological Inflexibility and Intolerance of Uncertainty Moderate the Relationship BETWEen Social Isolation and Mental Health Outcomes During COVID-19. JCBS (2020) 18:162–74. 10.1016/j.jcbs.2020.09.005 32953435 PMC7489247

[28] Warholm O Bjerkheim CR. Digital Mindsets, Job Satisfaction and the Role of Intolerance of Uncertainty: A Conditional Approach. Oslo: BI Norwegian Business School (2020). [master's thesis].

[29] Kabbash IA El-Sallamy RM Abdo SAEF Atalla AO. Job Satisfaction Among Physicians in Secondary and Tertiary Medical Care Levels. ESPR (2020) 27(30):37565–71. 10.1007/s11356-020-08506-9 32232753

[30] Spector PE. Job Satisfaction: Application, Assessment, Causes, and Consequences. California, United States: Sage (1997). 10.4135/9781452231549

[31] Hoboubi N Choobineh A Ghanavati FK Keshavarzi S Hosseini AA. The Impact of Job Stress and Job Satisfaction on Workforce Productivity in an Iranian Petrochemical Industry. SH@W (2017) 8(1):67–71. 10.1016/j.shaw.2016.07.002 28344843 PMC5355527

[32] Kaya F Oğuzöncül AF. Birinci Basamak Sağlık Çalışanlarında Iş Doyumu Ve Etkileyen Faktörler. Dicle Tıp Derg (2016) 43(2):248–55. 10.5798/diclemedj.0921.2016.02.0675

[33] Nal M Nal B. Sağlık Çalışanlarının Iş Doyumu Düzeylerinin Incelenmesi: Bir Kamu Hastanesi Örneği. ODÜSOBİAD (2018) 8(1):131–40.

[34] Kasemsap K. The Significance of Job Satisfaction in Modern Organizations. In: Christiansen B Chandan HC, editors. Handbook of Research on Human Factors in Contemporary Workforce Development. Pennsylvania: IGI Global (2017). p. 181–200. 10.4018/978-1-5225-2568-4.ch008

[35] Chao MC Jou RC Liao CC Kuo CW. Workplace Stress, Job Satisfaction, Job Performance, and Turnover Intention of Health Care Workers in Rural Taiwan. APJPH (2015) 27(2):NP1827–36. 10.1177/1010539513506604 24174390

[36] Larrabee JH Janney MA Ostrow CL Withrow ML Hobbs GR Burant C. Predicting Registered Nurse Job Satisfaction and Intent to Leave. JONA (2003) 33(5):271–83. 10.1097/00005110-200305000-00003 12792282

[37] Al-Ahmadi H. Factors Affecting Performance of Hospital Nurses in Riyadh Region, Saudi Arabia. Int J Health Care Qual Assur (2009) 22(1):40–54. 10.1108/09526860910927943 19284170

[38] Luthans F Youssef CM Avolio BJ. Psychological Capital: Developing the Human Competitive Edge. Oxford: Oxford University Press (2006). 10.1093/acprof:oso/9780195187526.001.0001

[39] Youssef‐Morgan CM Luthans F. Psychological Capital and Well-Being. Stress Health. (2015) 31(3):180–8. 10.1002/smi.2623 26250352

[40] Alat P Das SS Arora A Jha AK. Mental Health During COVID-19 Lockdown in India: Role of Psychological Capital and Internal Locus of Control. Curr Psychol (2021) 42:1923–35. 10.1007/s12144-021-01516-x 33746461 PMC7954522

[41] Newman A Ucbasaran D Zhu FEI Hirst G. Psychological Capital: A Review and Synthesis. J Org Behav (2014) 35(S1):S120–38. 10.1002/job.1916

[42] Abbas M Raja U Darr W Bouckenooghe D. Combined Effects of Perceived Politics and Psychological Capital on Job Satisfaction, Turnover Intentions, and Performance. J Manag (2014) 40(7):1813–30. 10.1177/0149206312455243

[43] Badran MA Youssef-Morgan CM. Psychological Capital and Job Satisfaction in Egypt. J Manag Psychol (2015) 30(3):354–70. 10.1108/JMP-06-2013-0176

[44] Luthans F Avolio BJ Avey JB Norman SM. Positive Psychological Capital: Measurement and Relationship With Performance and Satisfaction. Pers Psychol (2007) 60(3):541–72. 10.1111/j.1744-6570.2007.00083.x

[45] Mao Y He J Morrison AM Andres Coca-Stefaniak J. Effects of Tourism CSR on Employee Psychological Capital in the COVID-19 Crisis: From the Perspective of Conservation of Resources Theory. Curr Issues Tourism (2021) 24(19):2716–34. 10.1080/13683500.2020.1770706

[46] Koller SL Hicks RE. Psychological Capital Qualities and Psychological Well-Being in Australian Mental Health Professionals. Int J Psychol Stud (2016) 8(2):41–53. 10.5539/ijps.v8n2p41

[47] Çağış ZG Yıldırım M. Understanding the Effect of Fear of COVID-19 on COVID-19 Burnout and Job Satisfaction: A Mediation Model of Psychological Capital. Psychol Health Med (2023) 28(1):279–89. 10.1080/13548506.2022.2077970 35579863

[48] Mubarak N Safdar S Faiz S Khan J Jaafar M. Impact of Public Health Education on Undue Fear of COVID-19 Among Nurses: The Mediating Role of Psychological Capital. IJMHN (2021) 30(2):544–52. 10.1111/inm.12819 PMC7753350 33230850

[49] Liu L Chang Y Fu J Wang J Wang L. The Mediating Role of Psychological Capital on the Association Between Occupational Stress and Depressive Symptoms Among Chinese Physicians: A Cross-Sectional Study. BMC Public Health (2012) 12:219–8. 10.1186/1471-2458-12-219 22436106 PMC3410796

[50] Tian F Shu Q Cui Q Wang L Liu C Wu H. The Mediating Role of Psychological Capital in the Relationship Between Occupational Stress and Fatigue: A Cross-Sectional Study Among 1,104 Chinese Physicians. Front Public Health (2020) 8:12. 10.3389/fpubh.2020.00012 32185156 PMC7058796

[51] Yıldırım M Çağış ZG Williams G. Fear of COVID-19, Intolerance of Uncertainty, Psychological Capital, and Positive Future Expectations: Tests of Mediating Relationships With Healthcare Workers. Arch Psychiatr Nurs (2023) 45:158–63. 10.1016/j.apnu.2023.06.016 37544692 PMC10290176

[52] Vizheh M Qorbani M Arzaghi SM Muhidin S Javanmard Z Esmaeili M. The Mental Health of Healthcare Workers in the COVID-19 Pandemic: A Systematic Review. J Diabetes Metab Disord (2020) 19(2):1967–78. 10.1007/s40200-020-00643-9 33134211 PMC7586202

[53] Wu PE Styra R Gold WL. Mitigating the Psychological Effects of COVID-19 on Health Care Workers. CMAJ (2020) 192(17):E459–E460. 10.1503/cmaj.200519 32295761 PMC7207194

[54] Carleton RN Norton MPJ Asmundson GJ. Fearing the Unknown: A Short Version of the Intolerance of Uncertainty Scale. J Anxiety Disord (2007) 21(1):105–17. 10.1016/j.janxdis.2006.03.014 16647833

[55] Sarıçam H Erguvan FM Akın A Akça MŞ. The Turkish Short Version of the Intolerance of Uncertainty (IUS-12) Scale: The Study of Validity and Reliability. RESSJOURNAL (2014) 1(3):148–57. 10.17121/ressjournal.109

[56] Lorenz T Beer C Pütz J Heinitz K. Measuring Psychological Capital: Construction and Validation of the Compound PsyCap Scale (CPC-12). PLoS One (2016) 11(4):e0152892–17. 10.1371/journal.pone.0152892 27035437 PMC4817957

[57] Brayfield AH Rothe HF. An index of Job Satisfaction. J Appl Psychol (1951) 35(5):307–11. 10.1037/h0055617

[58] Judge TA Locke EA Durham CC Kluger AN. Dispositional Effects on Job and Life Satisfaction: The Role of Core Evaluations. J Appl Psychol (1998) 83(1):17–34. 10.1037/0021-9010.83.1.17 9494439

[59] Çöl G. Algılanan Güçlendirmenin Işgören Performansı Üzerine Etkileri. Doğuş Üniversitesi Derg (2008) 9(1):35–46. 10.31671/dogus.2019.220

[60] Karakum M. The Effects of Person-Organization Fit on Employee Job Satisfaction, Performance and Organizational Commitment in a Turkish Public Organization. Ankara: Middle East Technical University (2005). [master's thesis].

[61] Borman WC Motowidlo SJ. Expanding the Criterion Domain to Include Elements of Contextual Performance. In: Schmitt N Borman WC, editors. Personnel Selection in Organizations. NJ: Jossey-Bass (1993). p. 71–98.

[62] Befort N Hattrup K. Valuing Task and Contextual Performance: Experience, Job Roles, and Ratings of the Importance of Job Behaviors. Appl HRM Res (2003) 8(1-2):17–32.

[63] Hayes AF. Introduction to Mediation, Moderation, and Conditional Process Analysis: A Regression-Based Approach. New York: Guilford Press (2018).

[64] Preacher KJ Hayes AF. Asymptotic and Resampling Strategies for Assessing and Comparing Indirect Effects in Multiple Mediator Models. Behav Res Methods (2008) 40(3):879–91. 10.3758/BRM.40.3.879 18697684

[65] Lee S Jeon KO Kim H Chung EK. Effects of Intolerance of Uncertainty on Turnover Intention in Transplantation Coordinators: The Roles of Burnout and Grit. Korean J Transpl (2020) 34(4):265–71. 10.4285/kjt.20.0044 PMC9186814 35770109

